# Association Between Adjuvant Sorafenib and the Prognosis of Patients With Hepatocellular Carcinoma at a High Risk of Recurrence After Radical Resection

**DOI:** 10.3389/fonc.2021.633033

**Published:** 2021-09-23

**Authors:** Qingli Li, Tianqiang Song

**Affiliations:** Department of Hepatobiliary Cancer, Tianjin Medical University Cancer Institute and Hospital, Tianjin, China

**Keywords:** hepatocellular carcinoma, sorafenib, adjuvant therapy, hepatectomy, survival, recurrence

## Abstract

**Background:**

The use of sorafenib in the adjuvant management of hepatocellular carcinoma (HCC) is controversial.

**Aim:**

To analyze the effects of adjuvant sorafenib therapy in patients with HCC at high recurrence risk after radical resection.

**Methods:**

This was a retrospective study of patients who underwent radical resection (R0 resection) for HCC at the Cancer Hospital of Tianjin Medical University between August 2009 and August 2017. All patients had microvascular invasion and were evaluated for portal vein tumor thrombus. The outcomes were overall survival (OS), recurrence-free survival (RFS), and survival after recurrence. Propensity score matching (PSM) was used.

**Results:**

Before matching, there were 56 and 167 patients in the sorafenib and non-sorafenib groups. After PSM, there were 42 patients/group, and there were no significant differences in patient characteristics (all P>0.05). After PSM, compared with the non-sorafenib group, the sorafenib group showed longer median OS (34 *vs. *26 months, P=0.032) and survival after recurrence (16 *vs.* 9 months, P=0.002), but no difference in RFS (14 *vs.* 11 months, P=0.564). Adjuvant sorafenib was the only factor independently associated with OS (HR=0.619, 95% CI: 0377–0.994, P=0.047). No factors were independently associated with RFS (all P>0.05).

**Conclusion:**

Although adjuvant sorafenib therapy for patients with HCC and high recurrence risk does not reduce the recurrence risk of HCC, it might be associated with longer survival and a lower risk of death.

## Introduction

Hepatocellular carcinoma (HCC) is a highly lethal invasive carcinoma arising in the liver (1, 2). The most important risk factors for HCC are infection with hepatitis B or hepatitis C and/or preexisting liver cirrhosis (1–4). The incidence of HCC is higher in men and generally follows the geographical distribution of hepatitis B and C viruses (2–4). The worldwide age-standardized annual mortality rates of liver cancer are 13.9 per 100,000 men and 4.9 per 100,000 women (5). HCC is considered at a high risk of recurrence in the presence of vascular tumor thrombosis and a sum of lesion diameters >10 cm (3). The management of HCC is comprehensive and includes surgery, chemotherapy, targeted therapy, and radiation therapy (3). The prognosis of HCC is poor, with a 5-year overall survival (OS) of 18% for all-stage HCC (31% for localized disease, 11% for regional disease, and 3% for distant-stage disease) (6).

Sorafenib is an oral multitarget tyrosine kinase inhibitor that inhibits angiogenesis (7). It inhibits the vascular endothelial growth factor receptor (VEGFR), platelet-derived growth factor receptor (PDGF-R), Flt3, c-Kit, and Raf kinases (8, 9). It is considered standard-of-care for patients with unresectable HCC since 2007 (10). Sorafenib is expensive; some patients show no response, and there are adverse events (AEs) (7). At present, the indications of sorafenib are first-line treatment (10, 11) after arterially directed therapies and as bridge therapy for patients awaiting transplantation (12–16), but not as adjuvant therapy (3, 4).

The use of sorafenib in the adjuvant setting in patients at high risk of recurrence is controversial. The STORM phase III trial showed no difference between patients with HCC treated with adjuvant sorafenib or a placebo (17). On the other hand, a propensity score matching (PSM) analysis by Zhang et al. suggested that adding sorafenib after R0 HCC resection improved the OS and recurrence-free survival (RFS) in patients with microvascular invasion (18). Huang et al. showed that the use of sorafenib after curative hepatectomy in patients with HCC and microvascular invasion was independently associated with better OS and RFS (19). Similar results were reported by Zhuang et al. (20).

Hence, this study aimed to analyze the effects of adjuvant sorafenib therapy in patients with HCC at high recurrence risk after radical resection. The results could help improve the management of these patients.

## Materials and Methods

### Patients

This retrospective study included patients who underwent radical resection (R0 resection) for HCC at the Cancer Hospital of Tianjin Medical University from August 2009 to August 2017. This study was approved by the ethics committee of the Cancer Hospital of Tianjin Medical University. The requirement for informed consent was waived by the committee because of the retrospective nature of the study.

The inclusion criteria were 1) radical hepatectomy for HCC, 2) R0 resection, 3) microvascular invasion and high risk of recurrence, 4) evaluated for portal vein tumor thrombus (PVTT), 5) liver function grade A, and 6) Eastern Cooperative Oncology Group (ECOG) performance status (PS) score 0–1. The exclusion criteria were 1) intolerance to sorafenib within 2 weeks, 2) extrahepatic metastasis, 3) receiving other anti-cancer treatment before the operation, such as chemotherapy, radiotherapy, traditional Chinese medicine treatment, radiofrequency ablation, interventional therapy, targeted therapy, or immunotherapy, 4) another malignant tumor, 5) tumor thrombus in the main portal vein, or 6) liver function grading B or C. R0 resection was defined as complete removal of the tumor without residual, and no tumor found within one month after surgery (21). The patients were divided into the sorafenib and non-sorafenib groups according to whether they received postoperative sorafenib or not.

### Surgery and Adjuvant Therapy Post-Surgery

All patients underwent radical hepatectomy (R0 resection). All operations were performed by the same team of physicians. In selected patients, sorafenib was started within 1 month after the operation. All patients were prescribed sorafenib 400 mg twice a day. When grade 3 or 4 AEs (according to CTCAE) occurred, the dosage could be adjusted or the drug stopped until the AE was relieved or disappeared. Sorafenib was stopped when tumor progression occurred, or the AEs could not be tolerated, or there was an indication for drug termination.

The patients in the non-sorafenib group were treated conventionally and according to the guidelines (3, 4), using the current versions when the patients were treated. The adjuvant treatments included interventional therapy, biotherapy, ablation therapy, and radiation therapy.

### Data Collection and Definition

All data were collected from the hospital’s electronic medical record: sex, age, complications (hepatitis and cirrhosis), tumor size, tumor number, intrahepatic metastasis, tumor differentiation, satellite focus, *α*-fetoprotein (AFP), PVTT, TNM stage, and the outcome indicators (recurrence, RFS, RFS rate, OS, and survival after recurrence).

RFS was defined as the time from radical hepatectomy to recurrence or death. OS was defined as the time from radical resection to the date of death. Survival after recurrence was defined as the time from recurrence to death due to any cause. Follow-up was censored at the last follow-up in the absence of death or recurrence.

The follow-up data were from the patient charts. As per routine practice, during the first year after radical resection of HCC, routine blood tests, liver function tests, tumor markers (including AFP), and imaging examinations were performed every 1–2 months. Tumor markers (including AFP) and liver ultrasound were performed every 3 months during the second year. After 2 years, tumor markers (including AFP) and liver ultrasound were performed every 6 months, and upper abdomen plain and enhanced magnetic resonance imaging (MRI) were performed every 6–12 months. Patients with elevated levels of tumor markers or suspicious lesions by imaging were identified as high-risk populations, and they were reexamined with plain and enhanced MRI scanning. If necessary, positron emission tomography (PET)–computed tomography (CT) was performed. If a recurrence could not be determined, the patients continued to be closely followed.

Recurrence was defined as detecting definite recurrent cancer foci using the plain and enhanced MRI scanning of the upper abdomen. After the postoperative recurrence of HCC, individualized treatments (including resection, interventional therapy, radiofrequency ablation, antiviral therapy, targeted therapy, and immunotherapy) were selected according to the patient’s condition.

### Statistical Analysis

All statistical analyses were performed using SPSS 26.0 (IBM, Armonk, NY, USA). The continuous variables conforming to the normal distribution are presented as means ± standard deviation and were analyzed using Student’s t-test. Categorical variables are represented as n (%) and were analyzed using Fisher’s exact test. Survival was analyzed using the Kaplan–Meier method, and the curves were compared with the log-rank test. Cox regression analysis was used to analyze the factors influencing HCC recurrence, and a multivariable analysis was performed using variables with P-values ≤0.05 in the univariable analyses and variables with clinical significance. P-values <0.05 were considered statistically significant. The variables with significant differences were selected (sex, age, tumor size, tumor number, preoperative AFP level, tumor differentiation, hepatitis, cirrhosis, intrahepatic metastasis, satellite lesions, TNM stage, and resection of liver segments) and were used to match the patients 1:1 using PSM.

## Results

### Characteristics of the Patients

**Figure 1:**
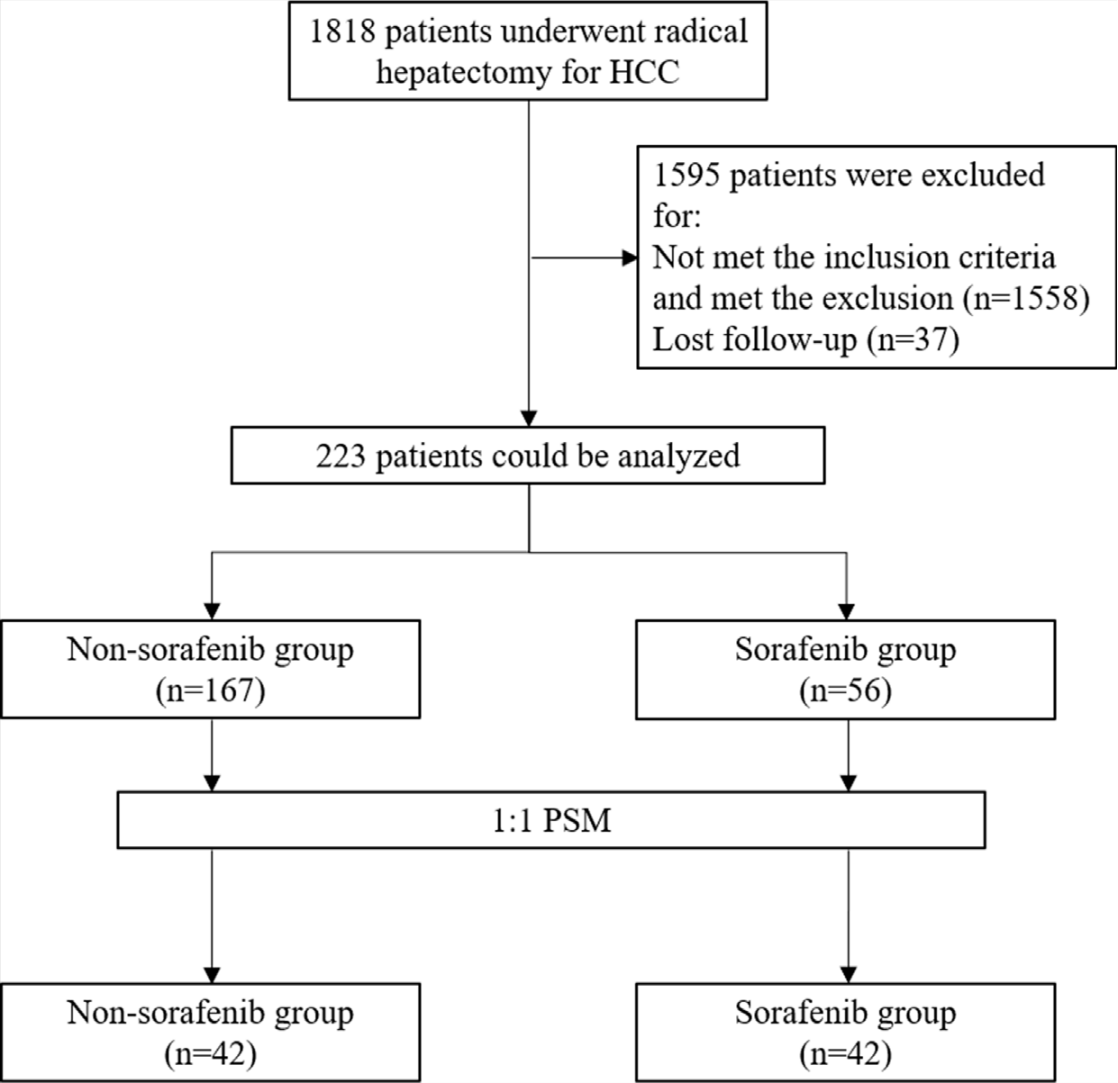
Flow chart.

A total of 1,818 HCC patients who underwent radical hepatectomy were screened. Before matching, there were 56 patients in the sorafenib group and 167 in the non-sorafenib group. After matching, there were 42 patients in each group (**Figure 1**). **Table 1** presents the characteristics of the patients. Compared with the non-sorafenib group, the patients in the sorafenib group had a lower frequency of hepatitis (69.6 *vs.* 88.6%, P = 0.001), higher frequency of cirrhosis (83.9 *vs.* 60.5%, P = 0.001), higher frequency of PVTT (21.4 *vs.* 7.2%, P = 0.004), and more advanced HCC stage (P = 0.005). After PSM, there were 42 patients in each group. There were no differences between the two groups for all variables (all P > 0.05).

**Table 1:** Clinical features before and after matching.

	Before matching			After matching		
	Sorafenib (n = 56)	Non-sorafenib (n = 167)	P	Sorafenib (n = 42)	Non-sorafenib (n = 42)	P
Sex, n (%)			0.14			0.776
Male	42 (75.0)	140 (83.8)		34 (81.0)	35 (83.3)	
Female	14 (25.0)	27 (16.2)		8 (19.0)	7 (16.7)	
Age (years, median ± SD)	54.4 ± 1.2	56.1 ± 0.9	0.238	54.2 ± 1.4	54.6 ± 1.7	0.573
Age, n (%)			0.399			0.826
>54 years	32 (57.1)	106 (63.5)		24 (57.1)	23 (54.8)	
<54 years	24 (42.9)	61 (46.5)		18 (42.9)	19 (45.2)	
Hepatitis, n (%)	39 (69.6)	148 (88.6)	0.001	33 (78.6)	34 (81.0)	0.786
Cirrhosis, n (%)	47 (83.9)	101 (60.5)	0.001	34 (81.0)	37 (88.1)	0.365
Tumor size (cm, median ± SD)	6.5 ± 0.6	5.5 ± 0.3	0.206	6.2 ± 0.6	7.2 ± 0.8	0.573
Tumor size, n (%)			0.058			0.113
>5 cm	32 (57.1)	70 (41.9)		23 (54.8)	30 (71.4)	
<5 cm	24 (42.9)	97 (58.1)		19 (45.2)	12 (28.6)	
AFP, n (%)			0.91			>0.99
>20 ng/ml	31 (55.4)	91 (54.5)		21 (50.0)	21 (50.0)	
<20 ng/ml	25 (44.6)	76 (45.5)		21 (50.0)	21 (50.0)	
Number of tumors, n (%)			0.83			0.459
Single	41 (73.2)	129 (77.3)		13 (31.0)	17 (40.5)	
Multiple	15 (26.8)	38 (22.7)		29 (69.0)	25 (59.5)	
PVTT, n (%)	12 (21.4)	12 (7.2)	0.004	7 (16.7)	9 (21.4)	0.505
Intrahepatic metastasis, n (%)	25 (44.6)	70 (41.9)	0.721	17 (40.5)	22 (52.4)	0.274
Tumor differentiation			0.501			>0.99
Low + moderate	18 (32.1)	62 (37.1)		12 (28.6)	12 (28.6)	
High	38 (67.9)	105 (62.9)		30 (71.4)	30 (71.4)	
TNM staging, n (%)			0.005			0.525
I	12 (21.4)	73 (43.7)		8 (19.1)	5 (11.9)	
II	28 (50.0)	69 (41.3)		22 (52.4)	21 (50.0)	
III	16 (28.5)	25 (15.0)		12 (28.6)	16 (38.1)	
Satellite lesions, n (%)	24 (42.9)	70 (41.9)	0.902	18 (42.9)	22 (52.4)	0.382
BCLC, n (%) (0/A/B/C)			0.31			0.71
0	5 (8.9)	14 (8.4)		5 (11.9)	2 (4.8)	
A	20 (35.7)	84 (50.3)		16 (38.1)	16 (38.1)	
B	19 (33.9)	56 (33.5)		14 (33.3)	15 (35.7)	
C	12 (21.4)	13 (7.8)		7 (16.7)	9 (21.4)	
Recurrence, n (%)	42 (75.0)	124 (74.3)	0.912	33 (78.6)	37 (88.1)	0.242
Death, n (%)	39 (69.6)	122 (73.1)	0.622	31 (73.8)	35 (83.3)	0.046

AFP, α-fetoprotein; PVTT, portal vein tumor thrombus; TNM, tumor-node-metastasis; BCLC, Barcelona Clinic Liver Cancer.

### Survival Analysis

Before PSM, there were no differences between the sorafenib and non-sorafenib groups for recurrence (75.0 *vs.* 74.3%, P = 0.912) and deaths (69.6 *vs.* 73.1%, P = 0.622). After PSM, there were no differences between the sorafenib and non-sorafenib groups for recurrence (78.6 *vs.* 88.1%, P = 0.242), but mortality was lower in the sorafenib group (73.8 *vs.* 83.3%, P = 0.046) (**Table 1**). In the non-sorafenib group, there was one case of lung metastasis, two of abdominal lymph node metastasis, one of brain metastasis, and one of bone metastasis. In the sorafenib group, there were two cases of lung metastasis and one of bone metastasis. There were no differences between the two groups before and after PSM (all P > 0.05). After recurrence, reoperation, interventional therapy, and radiofrequency ablation were mainly selected in the non-sorafenib group, but no targeted drugs such as sorafenib were used. In the sorafenib group, reoperation, interventional therapy, targeted drugs, radiofrequency ablation, and other adjuvant treatments were selected.

**Figure 2:**
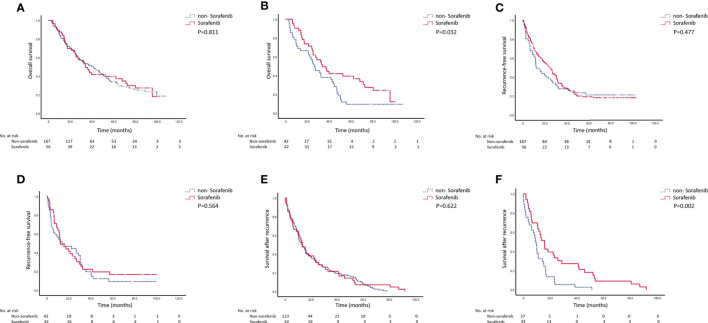
Recurrence-free survival (RFS), overall survival (OS), and survival after recurrence before and after matching. **(A)** OS before matching (P = 0.811). **(B)** OS after matching (P = 0.032). **(C)** RFS before matching (P = 0.477). **(D)** RFS after matching (P = 0.564). **(E)** Survival after recurrence before matching (P = 0.622). **(F)** Survival after recurrence after matching (P = 0.002).

**Figure 2** presents the survival analyses before and after PSM. There were no differences in OS between the two groups before PSM (P = 0.811). The sorafenib group’s median OS was 42 (95% CI: 30.5–52) months, and the 1-, 2-, and 3-year OS rates were 87.5, 67.9, and 47.5%, respectively. The median OS in the non-sorafenib group was 34.0 (95% CI: 27.5–40.5) months, and the 1-, 2-, and 3-year OS rates of the non-sorafenib group were 80.8, 65.3, and 53.0%, respectively. There were no significant differences between the two groups. After PSM, OS was better in the sorafenib group than in the non-sorafenib group (P = 0.032). The sorafenib group’s median OS was 34 (95% CI: 22.7–45.1) months, and the 1-, 2-, and 3-year OS rates of patients in the sorafenib group were 90.5, 71.4, and 49.5%, respectively. In the non-sorafenib group, the median OS was 26.0 (95% CI: 18.3–34.3) months, and the 1-, 2-, and 3-year OS rates were 69, 51.7, and 38.1%, respectively.

There were no significant differences between the two groups regarding RFS (P = 0.477). The RFS was 27 (95% CI: 20.3–29.9) months in the sorafenib group, and the 1-, 2-, and 3-year RFS rates were 87.5, 67.9, and 47.5%, respectively. The median RFS was 15 (95% CI: 6.5–18.1) months in the non-sorafenib group, and the 1-, 2-, and 3-year RFS rates were 80.8, 65.3, and 53.2%, respectively. After PSM, there were still no differences in RFS between the two groups (P = 0.564). The median RFS was 14 (95% CI: 10.1–30.5) months in the sorafenib group, and the 1-, 2-, and 3-year RFS rates were 54.3, 44.4, and 19.8%, respectively. The RFS was 11.0 (95% CI: 1.5–20.1) months in the non-sorafenib group, and the 1-, 2-, and 3-year RFS rates were 51.5, 37.3, and 22.3%, respectively.

There were no differences in survival after recurrence between the two groups before matching. The median post-recurrence survival was 13.6 (95% CI: 9.78–17.56) months in the sorafenib group and 13.4 (95% CI: 8.88–17.99) months in the non-sorafenib group (P = 0.622). After PSM, the survival rate after recurrence in the sorafenib group was significantly higher than in the non-sorafenib group (15.7 (95% CI: 11.75–19.78) *vs.* 9.4 (95% CI: 7.06–11.44), P = 0.002).

### Univariable and Multivariable Analyses After PSM

The univariable analyses in the PSM cohort showed that adjuvant sorafenib, tumor size, and TNM stage III were associated with OS, while adjuvant sorafenib was the only factor independently associated with OS (HR = 0.619, 95% CI: 0377–0.994, P = 0.047) (**Table 2**). As for RFS, the univariable analyses in the PSM cohort showed that satellite lesions were associated with RFS (P = 0.032) (**Table 3**).

**Table 2:** Univariable and multivariable analyses of survival in patients with HCC after radical resection in the PSM cohort.

Factors	OS			
	Univariable P	Multivariable analysis		
		HR	95%CI	P
Sex (male/female)	0.293			
Age (≥54/<54 years)	0.362			
Tumor size (≥5/<5 cm)	0.01	1.780	0.914–3.469	0.09
AFP (≥20/<20 ng/ml)	0.399			
Number of tumors (single/multiple)	0.339			
PVTT (yes/no)	0.959			
Intrahepatic metastasis (yes/no)	0.184			
Tumor differentiation (low + moderate/high)	0.285			
TNM staging (I/II/III)	0.049	1.088	0.568–2.083	0.433
Satellite lesions (yes/no)	0.520			
Sorafenib (yes/no)	0.047	0.612	0.377–0.994	0.047

OS, overall survival; HR, hazards ratio; CI, confidence interval; AFP, α-fetoprotein; PVTT, portal vein tumor thrombus; TNM, tumor-node-metastasis.

**Table 3:** Univariable analyses of recurrence in patients with HCC after radical resection in the PSM cohort.

Factors	Univariable analysis P
Sex (male/female)	0.257
Age (≥54/<54 years)	0.497
Tumor size (≥5/<5 cm)	0.088
AFP (≥20/<20 ng/ml)	1.000
Number of tumors (single/multiple)	0.436
PVTT (yes/no)	0.159
Intrahepatic metastasis (yes/no)	0.146
Tumor differentiation (low + moderate/high)	0.199
TNM staging (I/II/III)	0.284
Satellite lesions (yes/no)	0.032
Sorafenib administration (yes/no)	0.247

RFS, recurrence-free survival; HR, hazards ratio; CI, confidence interval; AFP, α-fetoprotein; PVTT, portal vein tumor thrombus; TNM, tumor-node-metastasis.

## Discussion

The use of sorafenib in the adjuvant management of HCC is controversial (17–20) and is currently not recommended by the guidelines (3, 4, 22). Therefore, this study aimed to analyze the effect of adjuvant sorafenib therapy in patients with HCC at high recurrence risk after radical resection. The results showed that adjuvant sorafenib therapy for patients with HCC and high recurrence risk did not reduce the recurrences of HCC but that it might be associated with longer survival time and lower risk of death.

Surgery is considered the only potentially curative treatment for HCC, but the recurrence rates remain high (23–25). Tumor invasion of the blood and lymphatic vessels, either as microinvasion or PVTT, is considered a marker of poor prognosis because tumor cells can easily detach and be blood-borne to distant locations (26–28). In this study, such patients with a poor prognosis were selected. Sorafenib is effective in patients with advanced HCC (11, 24), as well as in those with vascular microinvasion (17, 22, 29, 30). Sorafenib could be an interesting treatment option for managing microscopic tumor foci and disseminated tumor cells (20, 31).

In the present study, adjuvant sorafenib prolonged OS and survival after recurrence in patients with HCC at high risk of recurrence, but not the RFS. The main reason is probably that even though sorafenib does not delay relapse, since it is a targeted drug, it may not be able to play anti-angiogenesis and antitumor effects of cell proliferation if the tumors are small, but when the tumor reaches a certain size, its anti-angiogenesis effect comes into play, slowing down tumor growth, preventing tumor flare-up, and resulting in local control (32, 33). That is supported by other recent retrospective studies in similar patients (18–20, 34–36). In the study by Zhang et al., 113 patients could be matched in the sorafenib and non-sorafenib groups, and the adjuvant sorafenib group showed significantly longer OS and RFS, both before and after PSM (18). Huang et al. showed that RFS and OS were both longer with adjuvant sorafenib in a total of 49 patients (19). In a non-matched study, Zhuang et al. showed in 27 and 54 patients who did and did not receive sorafenib, respectively, that OS was longer with sorafenib than with surgery alone (20). On the other hand, the STORM trial (phase III) showed that adjuvant sorafenib could not improve OS in patients with HCC. The reason why the STORM trial was negative is probably that liver cancers with a moderate recurrence risk and even liver cancer with a low recurrence risk were included, making it impossible to separate the curves of the two groups. Nevertheless, the patients included in this study were all at high recurrence risk, so this study concludes that sorafenib extends OS in such patients. That is also supported by Li et al. and Xia et al. in patients with BCLC stage C HCC (34, 35). Wang et al. showed that sorafenib could prevent early HCC recurrence after surgery (36). The STORM trial is the only randomized controlled trial (RCT) on the subject (17). Although RCTs are considered to provide a higher level of evidence than retrospective studies, they usually have stringent inclusion criteria that often limit the generalizability of the results and limited follow-up periods. In addition, it has been suggested that the inconclusive results of the STORM trial could be due to the biological diversity of HCCs (37). Hence, additional RCTs should be performed to confirm the results.

In the present study, adjuvant sorafenib was independently associated with OS. That is supported by Huang et al. (19), who also showed that adjuvant sorafenib was the only factor independently associated with OS. They also showed that adjuvant sorafenib and Child-Pugh classification were independently associated with RFS, but the present study showed no factor associated with RFS. Those results support the Kaplan–Meier analyses.

This study has limitations. The sample size was small and from a single hospital. All patients with missing follow-up data were excluded. Our center is a tertiary cancer hospital with many cases being referred from distant or rural hospitals. These patients are usually discharged shortly after surgery to be followed in their living area. In addition, the patients with early-stage HCC were excluded because the inclusion criterion #3 was “microvascular invasion and a high risk of recurrence”. Therefore, there are selection biases to the present study. The patients’ characteristics were different, and PSM had to be used, but some patients with sorafenib could not be matched. In addition, subgroup analyses could not be performed because of the small number of patients. The longer OS in the sorafenib group might be related to the longer survival after recurrence, which might be caused by a slower progression of HCC after recurrence (38), but this will have to be examined in larger studies. Finally, the AEs could not be analyzed because of the possible under-reporting of grade 1–2 AEs and because some patients consulted other hospitals for grade 3–4 AEs.

In conclusion, although adjuvant sorafenib therapy for patients with HCC and high recurrence risk after radical resection does not reduce the recurrence risk of HCC, it might be associated with longer survival time and lower risk of death.

## Data Availability Statement

The original contributions presented in the study are included in the article/supplementary material. Further inquiries can be directed to the corresponding author.

## Ethics Statement

The studies involving human participants were reviewed and approved by the ethics committee of the Cancer Hospital of Tianjin Medical University. Written informed consent for participation was not required for this study in accordance with the national legislation and the institutional requirements.

## Author Contributions

QL contributed to design, analysis of data, drafted and critically revised the manuscript. TS participated in conception, design, and critically revised the manuscript. All authors contributed to the article and approved the submitted version.

## Conflict of Interest

The authors declare that the research was conducted in the absence of any commercial or financial relationships that could be construed as a potential conflict of interest.

## Publisher’s Note

All claims expressed in this article are solely those of the authors and do not necessarily represent those of their affiliated organizations, or those of the publisher, the editors and the reviewers. Any product that may be evaluated in this article, or claim that may be made by its manufacturer, is not guaranteed or endorsed by the publisher.
